# The Proteolytic Fraction From *Vasconcellea cundinamarcensis* Latex Displays Anti-Inflammatory Effect in A Mouse Model of Acute TNBS-Induced Colitis

**DOI:** 10.1038/s41598-020-59895-3

**Published:** 2020-02-20

**Authors:** Ronniel Morais Albuquerque, Marina Passos Pizzitola, Ana Cândida Araújo e Silva, Dalton Dittz, Kátia Michelle Freitas, Ênio Ferreira, Carlos Edmundo Salas, Miriam Teresa Paz Lopes

**Affiliations:** 1 Pharmacology Department, Federal University of Minas Gerais, Belo Horizonte, Minas Gerais Brazil; 2 Mucuri Medicine School, Federal University of Jequitinhonha and Mucuri Valleys, Teófilo Otoni, Minas Gerais Brazil; 3 Biochemistry and Pharmacology Department, Federal University of Piauí, Teresina, Piauí Brazil; 4 Pathology Department, Federal University of Minas Gerais, Belo Horizonte, Minas Gerais Brazil; 5 Biochemistry and Immunology Department, Federal University of Minas Gerais, Belo Horizonte, Minas Gerais Brazil

**Keywords:** Proteases, Pharmacodynamics

## Abstract

The proteolytic fraction (P1G10) from *Vasconcellea cundinamarcensis*, displays gastric protective and healing activities in different skin lesions in mice and human. In an excisional model, this fraction accelerates resolution of lesions and modulates inflammatory mediators. Based on these data, we assessed its anti-inflammatory activity in murine colitis model, induced by 2,4,6-trinitrobenzenesulfonic acid (TNBS) adopted by its physiopathological similarity with human colitis. Twenty four hours after colitis induction followed by three days of treatment, P1G10 at 0.3 and 3.0 mg/Kg induced 30% increase in body weight (p < 0.0001) and ~80% reduction in colon macroscopic damage score (p < 0.05) compared to the untreated TNBS-induced colitis group. Histological analyses showed that 0.3 mg/Kg P1G10 reduced the inflammatory profile and tissue damage (47%, p < 0.05) when it was proteolytically active. Compared to TNBS group, 0.3 mg/Kg P1G10 reduced MPO activity (80%, p < 0.01), MCP-1 (47%, p < 0.05) and TNF-α (50%, no significant) and increased IL-10 (330%, p < 0.001) levels in the supernatant of colonic tissue homogenate. P1G10 treatment also reduced COX-2 expression (60%, p < 0.05) and metalloprotease-2 activity (39%, p < 0.05) while increased globet cell density (140%, p < 0.01), that contributes to mucus layer protection in colonic tissue. Taken together, these findings suggest that low doses of active P1G10 promotes lesion resolution, at least in part by its anti-inflammatory activity, in TNBS-colitis model.

## Introduction

Our group has studied the proteases from *Vasconcellea cundinamarcensis* latex, ethnopharmacologically used to treat digestive disorders and to enhance wound healing^1^ and later, biochemically characterized^2,3^. The protein fraction (P1G10) is obtained after removal of insoluble material from latex; it is composed of 14 proteolytic isoforms, sequentially activated after fruit excision^4,5^. P1G10 displays gastric protective and healing activities by increasing mucus content and PGE2 secretion, in indomethacin and acetic acid induced rodent lesions^6^. P1G10 also reduced gastrin levels, preventing hyperacidity and gastric damage^7^ and improved repair in excisional wounds, increasing the lesion resolution and modulating inflammatory mediators^8^. In this excisional model the proteolytic activity of P1G10 was required to enhance healing, while it was not relevant for gastroprotective action^7^.

The inflammatory bowel disease (IBD) encompasses a family of chronic, idiopathic, relapsing, and tissue-destructive conditions characterized by altered cytokine production and cellular inflammation, leading to prolonged and occasionally irreversible damage and integrity of gastrointestinal function^9^. Its aetiology is still obscure, but it seems to result from complex interactions between genetic predisposition, microbial factors and the immune system. Human IBDs are grouped into two major phenotypes: Crohn’s disease and ulcerative colitis^10^.

Both, the inflammatory process in IBDs and the 2,4,6-trinitrobenzenesulfonic acid (TNBS) colitis model share an imbalance between pro- and anti-inflammatory cytokines, with significant increase in IL-1, IL-6, TNF-α and IFN-γ^11,12^. The reduction of anti-inflammatory interleukin, IL-10 is singled out as main aggravating factor in IBDs and there is evidence that an increase in IL-10 prevents inflammation and mucosal damage in human colitis^11,13^. Neutrophils, the most abundant cells found in acute TNBS model and human colitis, play a key role during tissue damage, by releasing free radicals^14^. Mucosal integrity is disrupted, leading to a persistent inflammatory stimulus by luminal antigens^15^. Furthermore, it is recognized that cyclooxygenase-2 (COX-2) and metalloproteases (MMP) levels are elevated in injured GI sites contributing to development and progress of inflammation^16,17^.

Based on the anti-inflammatory properties of P1G10 from *V cundinamarcensis*, we propose investigating the role of this fraction on TNBS induced IBDs mice model, and analyse the possible participation of inflammatory markers.

## Materials and Methods

### Latex collection and fractionation of P1G10

Unripe fruits of *Vasconcellea cundinamarcensis* were the source of latex collected by longitudinal incisions onto the surface fruits with the aid of a sharp blade. After collection into plastic dark container, latex was stored in the dark at −20 °C until lyophilized. Dried latex was suspended in buffer containing 25 mM L-cysteine, 5 mM DTT and 10 mM EDTA pH 5.0 in 1 M sodium acetate solution and chromatographed on Sephadex-G10 as described before^6^. The first protein fraction emerging from the column containing the bulk proteolytic activity (P1G10) was pooled and concentrated by ultrafiltration (10,000 Da pore size) and stored at −20 °C until use. The protein concentration and amidase activity in P1G10 stocks used in this study were 8.46 ± 1.60 mg/ml and 29.09 nm/min/mg, respectively. Proteolytic activity in P1G10 was inhibited by incubation of 0.2 mM P1G10 for 5 min at room temperature with 2 mM iodoacetamide (IAA) in the presence of 1 mM cysteine. After extensive dialysis, the residual proteolytic activity (≤3%) was assessed using substrate D,L-BAPNA^5^.

### Animals

Male Swiss mice (30–35 g), obtained from CEBIO (ICB/UFMG) were housed at 22 ± 2 °C under a 12/12 h light/dark cycle. The animals were food-fasted 24 h before experimental procedures and allowed food and water *ad libitum* after the induction of lesions.

### Lesion induction and treatment

Animal procedures have been previously approved by the Federal University of Minas Gerais Ethics Committee (#177/2013). All experiments were performed in accordance with relevant guidelines and regulations. The colonic lesions were induced in 24 h-fasted mice by using a technique of hapten-induced colonic inflammation described by Antoniou *et al*.^12^. TNBS (1.5 mg/mouse) was dissolved in 50% ethanol (v/v) and injected (0.2 mL) into the colon using a rubber cannula, inserted at the splenic flexure (4 cm from the anus) in anesthetized mice (xylazine 10 mg/Kg and ketamine 80 mg/Kg). A Sham group received saline solution by same route. Animals were then kept for 2 min in vertical position and returned to their cages. After 24 h, mice treated with TNBS were allocated into groups of 8–11 animals and orally treated once a day, with saline (control group), P1G10 (0.3–30 mg/Kg) or proteolytically inhibited P1G10 (P1G10-IAA −0.3 mg/Kg) for three days (days 2–4). The Sham group was also treated with saline. By the end of treatment, mice were sacrificed in a CO_2_ chamber and a 5 cm colon segment was excised for macroscopic evaluation. Tissue segments were then fixed in 10% buffered formalin or immediately frozen in liquid nitrogen and stored at −70 °C for histological, immunohistochemical and biochemical studies.

### Macroscopic colonic damage analysis

After dissection, the colon was gently rinsed with saline solution, opened thru a longitudinal incision and photographed (Sony Cybershot DSC-W800). Visible colonic damage was analysed by a blinded participant who applied a semi-quantitative scoring method^18,19^. The morphological criteria in Table 1 summarize the symptoms under analysis.

**Table 1:** Macroscopic damage score.

Score	Damage criteria
0	Absence of injuries.
1	Hyperemia without ulcerations.
2	Hyperemia and local bowel wall thickening, without ulcerations.
3	Unifocal ulceration <0.5 cm.
4	Multifocal ulcerations <0.5 cm each; and/or an inflammation focus <1 cm.
5–6	Inflammation >1 cm. One point increased to the presence of ulceration sites <0.5 cm.
6–10	Ulceration site >0.5 cm. One point increased to each 0.5 cm of damage (hyperemia, ulceration or inflammation sites). Limited to a maximum of 10 points.
+0 or 1	Absence or presence of rectal prolapse.

Adapted^18,19^.

### Histopathological analysis

For histological evaluation, formalin-fixed tissue sections (5 μm) were embedded in paraffin and stained with haematoxylin and eosin and evaluated by light microscopy by a pathologist blinded to the experimental protocol. Morphometric analysis of colon fragments diagnosed as inflamed (score A) and intestinal structure (score B) were assessed according to the criteria shown on Table 2 ^20^. Histological total damage score was given by the sum of scores from A and B. We also performed periodic acid-schiff (PAS) staining method for histological light microscopy evaluation of goblet cell number and intestinal mucus layer integrity, performed by a non-participant pathologist.

**Table 2:** Histological damage score.

Inflammatory cell infiltrate		Score A	Intestinal architecture		Score B
Severity	Extent		Epithelial changes	Mucosal architecture	
Mild	Mucosa	1	Focal erosions		1
Moderate	Mucosa and submucosa	2	Erosions	±Focal ulcerations	2
Marked	Transmural	3	—	Extended ulcerations ± granulation tissue ± pseudopolyps	3

Adapted^20^.

### Neutrophil infiltration and macrophage activation

Neutrophil activation in colon samples was measured by myeloperoxidase (MPO) activity as previously described^21^. Sham, TNBS and 0.3 mg/Kg P1G10 tissue samples were weighed, homogenized in pH 4.7 buffer (0.1 M NaCl, 0.02 M NaPO_4_, 0.015 M EDTA), centrifuged at 12,000 × *g* for 10 min. The pellets were then resuspended in 0.05 M NaPO_4_ buffer (pH 5.4) containing 0.5% hexadecyltrimethylammonium bromide (HTAB) followed by three freeze-thaw cycles in liquid nitrogen. MPO activity in the supernatant was measured by the change in absorbance at 450 nm using 1.6 mM tetramethylbenzidine and 0.3 mM H_2_O_2_ substrates. The reaction was stopped with 50 μL of 4 M H_2_SO_4_. Results were expressed as change in absorbance/g wet tissue.

Mononuclear cell infiltration into the colon was indirectly measured by dosage of lysosomal N-acetylglucosaminidase (NAG) activity present in high levels in activated macrophages^22^. Tissue samples from Sham, TNBS and 0.3 mg/Kg P1G10 were homogenized in 0.9% NaCl solution (w/v) containing 0.1% Triton X-100 (v/v) (Promega) and centrifuged (3,000 × *g*; 10 min at 4 °C). Aliquots (100 μL) of supernatant were incubated for 10 min with 100 μL of p-nitrophenyl-N-acetyl-beta-d-glucosaminide (Sigma) dissolved in citrate/phosphate buffer (0.1 M citric acid, 0.1 M Na_2_HPO_4_; pH 4.5) to yield a final concentration of 2.24 mM. The reaction was stopped with 100 μL of 0.2 M glycine buffer (pH 10.6). Hydrolysis of the substrate was determined by measuring the absorption at 400 nm. Results were expressed as change in absorbance/g wet tissue.

### Cytokines levels

Colon samples were homogenized in PBS pH 7.4 containing 0.05% Tween and centrifuged at 10,000 × *g* for 30 min. TNF-α, MCP-1 and IL-10 were measured in 50 μL aliquots of supernatant using Immunoassay Kits (R&D Systems, USA) according to the manufacturer’s protocol. Briefly, dilutions of cell-free supernatants were added in duplicate to ELISA plates coated with a specific murine monoclonal antibody against the cytokine, followed by the addition of secondary horseradish peroxidase-conjugated polyclonal antibody. After washing to remove any unbound antibody-enzyme reagent, a substrate solution (50 μL of a 1:1 solution of H_2_O_2_ and 10 mg/mL tetramethylbenzidine dissolved in DMSO) was added to the mix. The reaction was stopped after 20 min incubation, with 2 N sulfuric-acid (50 μL) and the reaction rate measured at 540 nm. The results were expressed as pg cytokine/mg wet tissue.

### Gelatin zymography

Gelatinolytic activity of matrix metalloproteinase 2 (MMP-2, 72 kDa type IV collagenase or gelatinase A) and metalloproteinase 9 (MMP-9, 92 kDa type IV collagenase or gelatinase B) were assayed using 0.1% gelatine zymographic analysis previously described^23^. Aliquots of supernatants, obtained for NAG evaluation (5 μL), were incubated with 4.2 μL sample buffer (63 mM Tris, pH 6.8, 2% SDS, 10% glycerol, 0.0025% bromophenol blue) for 10 min at room temperature. Then, the samples were electrophoresed through 10% SDS-PAGE gels containing 0.1% gelatine, at 90 V. Removal of SDS was done by gel washing with 2.5% Triton X-100 for 1 h at room temperature followed by overnight incubation in buffer (50 mM Tris-HCl, pH 7.6, containing 10 mM CaCl_2_ and 0.15 M NaCl). After gel staining with Coomassie blue R-250 and distaining with a methanol-acetic acid mix, the gelatinolytic activity was displayed as clear bands over a deep blue Coomassie background. Molecular mass standards (BioRad Laboratories, Hercules, CA) were electrophoresed in parallel with samples to assess protein size. Digital images and densitometric analyses of the gels were obtained with ImageQuant® LAS4000 (GE-Healthcare®), and the MMPs activity was quantified with ImageQuant® software.

### Western blot assay

Tissue lysates were obtained from dissected samples stored at −80 °C. Colon tissue fragments were immersed in liquid nitrogen, triturated and then homogenized with potassium phosphate buffer (0.1 M, pH 6.5). The samples were then centrifuged at 13,000 × *g* for 10 min at 4 °C to pellet cell debris and the protein concentration in the supernatant lysate was measured by the Bradford method. Tissue lysates samples containing 30 μg of protein were loaded onto a 12% SDS-PAGE on a Bio-Rad Mini PROTEAN Tetra System (Bio-Rad Laboratories Inc) and then semi-dry electro transferred (1.0 A for 30 min) onto nitrocellulose membrane previously activated with methanol. Tris-buffered saline (TBS) with 5% BSA containing 0.1% Tween-20 was used to block the membrane at 4 °C for 1–2 h. Later, the membrane was incubated with COX-2 (1:200, Abcam) or β-actin (1:1000, Cell Signalling Technology, Inc.) primary antibody overnight at 4 °C. Then, the membrane was incubated with secondary antibody conjugated with peroxidase at 25 °C for 2 h. Development was done with Luminatta Forte (Millipore®) and digital images were analysed in ImageQuant® LAS4000 (GE-Healthcare®).

### Statistical analyses

Statistical analysis was done with Graph Prism software (GraphPad Software Inc., La Jolla, California, USA). The results are expressed as mean ± standard error of the mean. Parametric data were analysed by one-way ANOVA analysis of variance followed by the Newman–Keuls test to compare three or more groups. Body weight was analysed by two-way ANOVA followed by Bonferroni post-test. Non-parametric data were analysed by Kruskal-Wallis analysis followed by the Dunn’s post-test to compare three or more groups. Statistical significance was set at p < 0.05.

## Results

### P1G10 prevents body weight loss in TNBS-induced colitis

**Figure 1:**
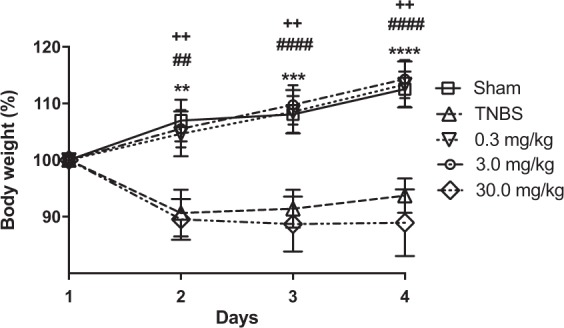
Body weight changes after TNBS treatment. Colitis was induced with TNBS (day 1) and animals were orally treated with P1G10 0.3, 3.0 or 30.0 mg/Kg or saline during days (days 2^nd^ thru 4^th^). Mean body weight changes were expressed as a percentage of each group relative to weight on day 1^st^ (n = 4–9). Statistical analysis was done by two-way ANOVA and Bonferroni post-test comparison with TNBS group: ^++^Sham, p < 0.01); **, ***, ****P1G10 0.3 mg/Kg, p < 0.01, p < 0.001 and p < 0.0001, respectively); ^##^, ^####^P1G10 3.0 mg/Kg, p < 0.01 and p < 0.0001, respectively).

A common symptom associated with IBD is weight loss due to malabsorption, food aversion or anorexia. We monitored body weight following TNBS treatment and changes were expressed as % mean weight of each group relative to day-first of treatment (Fig. 1). The TNBS group had a significant reduction in body weight (0.83-fold, on day 4^th^; p < 0.01), like in the group treated with 30 mg/Kg P1G10. On that day, Sham, P1G10 0.3 and 3.0 mg/Kg groups had similar weights, 1.13-fold (p < 0.01), 1.13-fold (p < 0.001) and 1.14-fold (p < 0.001), respectively, relative to the TNBS group (Fig. 1). The rationale for using this dose range is that in animal model, oral P1G10 ≥ 100 mg/Kg diminishes the protection of indomethacin induced gastric lesions^6^, while at 0.1 mg/Kg P1G10 there was no visible effect (not shown).

### P1G10 reduces colonic damage in TNBS-induced colitis

**Figure 2:**
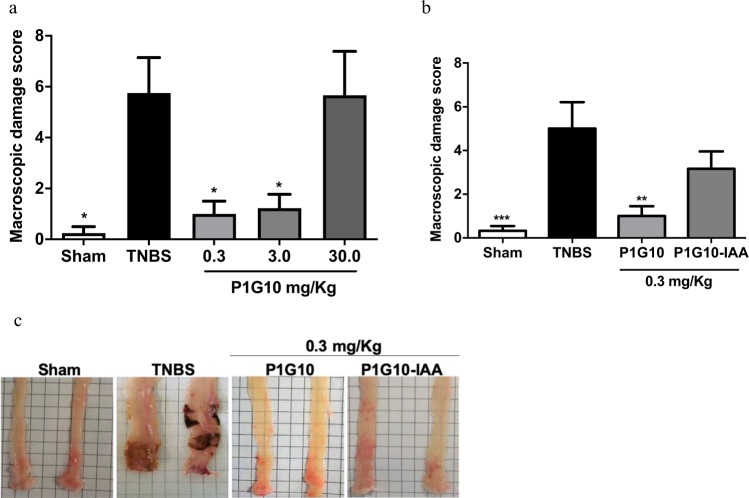
Effect of P1G10 in TNBS-induced colonic damage in mice. Colitis was induced with TNBS on day 1 and animals were treated with P1G10 (0.3, 3.0, 30.0 mg/Kg), P1G10-IAA 0.3 mg/Kg or saline during three consecutive days (days 2–4). After sacrifice on 4^th^ day, colon damage was scored in colon sections (**a**) P1G10 effect (n = 4–9) and (**b**) proteolytically inhibited P1G10 (n = 6–7). (**c**) Representative opened colon segment of: Sham, TNBS and P1G10 (0.3 mg/Kg) group. Statistical analysis by Kruskal-Wallis and Dunn’s post-test compared with TNBS group: *, **p < 0.05 and p < 0.01, respectively).

The microscopic damaged scored elicited by P1G10 in TNBS groups showed reduction of colonic damage (Fig. 2a) at 0.3 and 3.0 mg/Kg (83% and 79%, respectively, p < 0.05), while at 30 mg/Kg P1G10 there was no observable difference relative to the TNBS group. Therefore, the lower dose and the equivalent dose devoid of proteolytic activity (P1G10-IAA) were next chosen to assess the relevance of the proteolytic effect. As shown in Fig. 2b, only P1G10 at 0.3 mg/Kg reduced the macroscopic score damage compared to TNBS group (80%, p < 0.05). Meanwhile the decreased in damage observed at 0.3 mg mg/Kg P1G10-IAA was not significant. Fig. 2c shows representative images of damaged colonic segments after induction of colitis and subsequent treatments. Based on these results showing the anti-inflammatory action at 0.3 mg/Kg, subsequent analyses were done using this dose.

### Histological analysis

**Figure 3:**
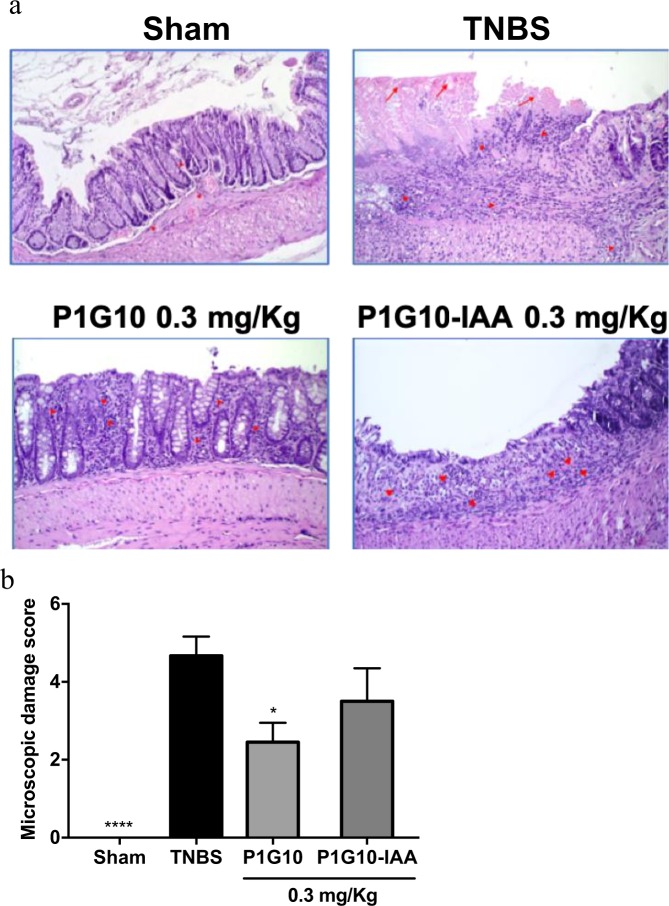
Histological analysis of P1G10 effect and relevance of its proteolytic action on TNBS-induced colitis. Colitis was induced with TNBS and animals were orally treated with P1G10 0.3 or 3.0 mg/Kg or saline for three days. After sacrifice on day 4^th^, colonic fragments were dissected, fixed and stained. (**a**) Red arrows: necrosis and ulceration; Red arrows head: inflammatory infiltrate. Magnification: 200x. (**b**) Microscopic analysis is shown as score, and evaluated as described in method section (n = 6–12). Statistical analysis by Kruskal-Wallis and Dunn’s post-test comparing with TNBS group: *, ****p < 0.05 and p < 0.0001, respectively).

Histological analysis shows that TNBS induced mucosal erosion and ulceration, submucosal oedema and occasionally transmural inflammation (Fig. 3a –upper panel). The presence of 0.3 mg/Kg P1G10 decreased inflammation and damage severity (Fig. 3a lower panel). Morphometric analysis of colon tissue stained with HE was used to highlight inflammatory structural parameters, annotated as microscopic score (Table 2). Figure 3b shows a reduction (47%, p < 0.05) of the inflammatory profile and tissue damage in animals treated with 0.3 mg/Kg P1G10 compared to TNBS group. Although 0.3 mg/Kg P1G10-IAA reduced the microscopic score, there was no statistical difference with TNBS group.

### P1G10 inhibits infiltration of inflammatory cells in TNBS-induced colitis

**Figure 4:**
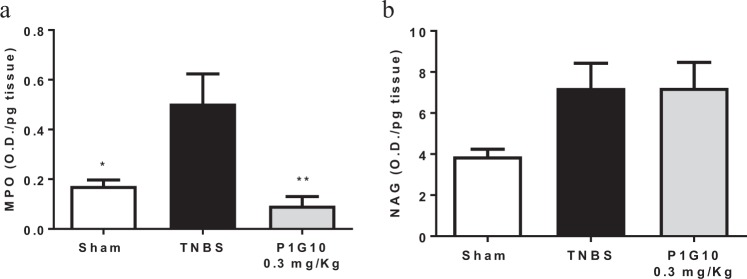
MPO and NAG activity in colonic tissue. Homogenates from colonic specimens were assayed by ELISA. (**a**) Myeloperoxidase (MPO) and (**b**) N-acetyl-beta-D-glucosaminidase (NAG) activities as index of neutrophil infiltration (n = 4–8) and macrophage activity (n = 4–8), respectively. Statistical analysis by ANOVA and Student-Newman-Keuls post-test comparison with TNBS group: *, **p < 0.05 and p < 0.01, respectively).

Figure 4 shows the MPO and NAG profiles on day fourth post TNBS induction. MPO activity, an indicator of neutrophil infiltration, increased significantly in TNBS group compared with Sham (p < 0.05). P1G10 0.3 mg/Kg decreased MPO activity 5.4-fold compared to the TNBS group (p < 0.01), attaining levels similar to Sham (Fig. 4a). Meanwhile the non-significant increase in NAG (77%) induced by TNBS, attributed to macrophage infiltration was not reversed in the group treated with P1G10.

### P1G10 re-establishes the balance between pro and anti-inflammatory cytokines

**Figure 5:**
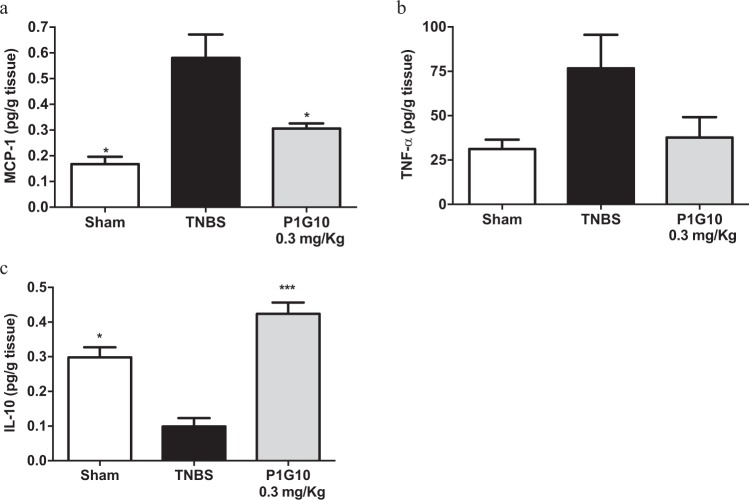
Effect of P1G10 on MCP-1, TNF-α and IL-10 in colonic mucosa from TNBS-induced colitis. Homogenates from colonic specimens were assayed by ELISA for cytokine level; (**a**) MCP-1 (n = 3–6); (**b**) TNF-α (n = 3–5); (**c**) IL-10 (n = 3–6). Statistical analysis by ANOVA and Student-Newman-Keuls post-test comparison with TNBS group: *, ***p < 0.05 and p < 0.001, respectively).

The level of pro-inflammatory (TNF-α, MCP-1) and anti-inflammatory (IL-10) cytokine were measured to analyse the influence of P1G10 as a potential regulator of inflammatory pathways (Fig. 5). In supernatants from TNBS colon, MCP-1 declined 47% after treatment with 0.3 mg/Kg P1G10, compared to the TNBS group (0.31 ± 0.02 *versus* 0.58 ± 0.09; p < 0.05). A similar reduction in TNF-α was observed 50%; (37.73 ± 11.46 *versus* 76.74 ± 18.82), but, it was not significant (Fig. 5b). Also, in the TNBS-group, IL-10 declined 67%, (p < 0.05) compared to Sham group, and upon treatment with 0.3 mg/Kg P1G10 IL-10 became higher than the Sham group (p < 0.001, Fig. 5c). The rise in IL-10 by 0.3 mg/Kg P1G10 was 42% higher than Sham group, but this difference was not significant.

### P1G10 reduces COX-2 level in TNBS-induced colitis

**Figure 6:**
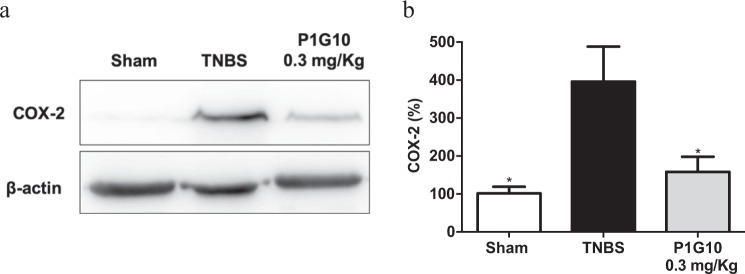
Effect of P1G10 on COX-2 in colonic tissue from TNBS-induced colitis. (**a**) COX-2 immunoblot from representative colonic tissue in Sham, TNBS and 0.3 mg/Kg P1G10; β-actin used as control (n = 3). (**b**) Densitometric analysis of immunoblots against COX-2 (n = 6–7). Statistical analysis by ANOVA and Student-Newman-Keuls post-test comparison with TNBS group: *p < 0.05).

To confirm the anti-inflammatory role of P1G10, the level of COX-2 was analysed by WB and densitometry in TNBS and P1G10 treated groups. Compared to Sham, COX-2 level was 3.9-fold higher in TNBS group (p < 0.05). P1G10 reduced COX-2 by 60% relative to the TNBS group (p < 0.05), attaining a value similar to the Sham group (Fig. 6). Individual blots for COX-2 and β-actin can be found as Supplementary Fig. S1.

### P1G10 decrease levels of MMP-2 and MMP-9 in TNBS-induced colitis

**Figure 7:**
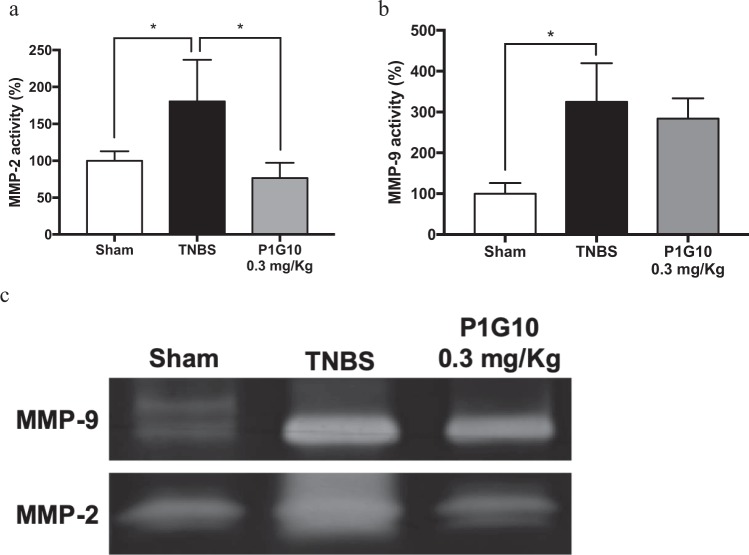
Effect of P1G10 on MMP-2 and MMP-9 activity in colonic mucosa from mice treated with TNBS. Homogenates from colonic specimens were assayed for (**a**) MMP-2 (n = 3–5) and (**b**) MMP-9 (n = 3–5) by zymography. (**c**) Representative zymographies of colonic tissue from Sham, TNBS and 0.3 mg/Kg P1G10 samples. Results were expressed as percentage of Sham media. Statistical analysis by ANOVA and Student-Newman-Keuls post-test comparison with TNBS group: *, **p < 0.05 and p < 0.01, respectively).

The activities of inflammatory metalloproteases MMP-2 and MMP-9, were measured by zymography and compared to the Sham group. MMP-2 (Fig. 7a) and MMP-9 (Fig. 7b) increased in TNBS groups (p < 0.05). On the other hand, 0.3 mg/Kg P1G10 decreased MMP-2 activity by 23% relative to the TNBS group (p < 0.05). A similar tendency was observed for MMP-9 activity, but without statistical significance. Individual gels for MMP-9 and MMP-2 can be found as Supplementary Fig. S2.

### P1G10 increase PAS positive cells in TNBS-induced colitis

**Figure 8:**
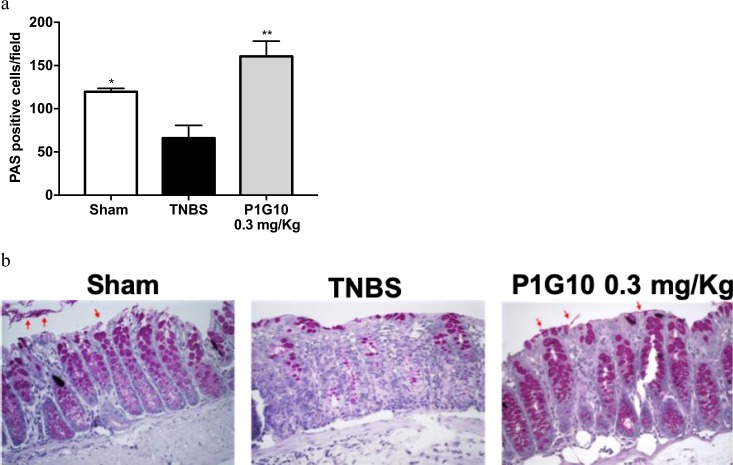
PAS analysis of P1G10 effect on TNBS-induced colitis. Colitis was induced with TNBS and 24 h after; animals were treated with 0.3 mg/Kg P1G10 during 3 days. After sacrifice on day 4^th^, colonic fragments were dissected, and sections fixed and stained. (**a**) Microscopic analysis is shown as number of positive PAS cells per field (n = 3–6). (**b**) Red arrows: intestinal mucus layer. Magnification: 400x. Statistical analysis by ANOVA and Student-Newman-Keuls post-test comparison with TNBS group: *, **p < 0.05 and p < 0.01, respectively).

The PAS staining method was applied for assessment of goblet cells and their integrity in intestinal mucus layer, a primary barrier for the protection of the intestine. Compared to Sham, TNBS group decreased goblet cells by 55% (p < 0.05). In TNBS group, it was observed that the mucus layer was absent in some inflamed sites. Treatment with P1G10 increased 2.4-fold the number of goblet cells compared to TNBS group (p < 0.01, Fig. 8a), demonstrating a protective effect on the intestinal mucous layer. Figure 8b show sections of the distribution of goblet cells and mucus layer after colitis induction with and without treatment with P1G10.

## Discussion

As previously demonstrated, P1G10 is a proteolytic fraction from *V*. *cundinamarcensis* latex displaying healing activity in wound skin models and gastric mucosal lesions^6,7,24,25^. Considering the aetiology and clinical importance of inflammatory bowel disease (IBDs), this study shows the beneficial effect of P1G10 on an intestinal injury model induced by 2,4,6-trinitrobenzenesulfonic acid (TNBS). In the TNBS model, GI inflammation results from covalent attachment of the haptenizing agent to endogenous proteins of host mucosa, developing an extended hypersensitive stimulus to TNBS-modified antigens^26^. This interaction produces focal oedema, ulcers, loss of epithelial integrity, mucosal and submucosal infiltration of inflammatory cells and granuloma^27^. The TNBS model has been commonly used for screening potential drugs as treatment for ulcerative colitis and colon damage^28,29^.

A common symptom associated with IBD is weight loss, mostly due to malabsorption, anorexia and uncontrolled diarrhea^30^. When assessing body weight change after TNBS induction and subsequent treatments, we observed that P1G10 at 0.3 or 3.0 mg/Kg prevented weight loss (Fig. 1). Weight loss, diarrhea and rectal prolapse symptoms result from permeability changes in intestinal mucosa due to structural and functional damage induced by TNBS^31^. In addition, macro and microscopic analysis of TNBS colonic lesions (Figs. 2 and 3, respectively) displayed reduced inflammatory pattern and reduced damage in presence of 0.3 mg/Kg P1G10. In contrast, at 30.0 mg/Kg, the protection against weight-loss disappeared (Fig. 1). A similar effect was seen in indomethacin-induced gastric ulcers, where above 10 mg/Kg, P1G10 lost ulcer protective efficacy^6^. The presence of various types of proteases is central to the physiology of GI tract and their expression is increased during IBD, in association with the inflammatory process^32–34^. In this scenario, the role of P1G10 can be theorized assuming that plant proteases display substrate specificity oriented to a defensive role in latex containing plants^35^. Thus, in the GI tract, exogenous proteases, may act as anti-inflammatories by inhibiting or potentially competing with host inflammatory proteases for their cognate substrates. During pyloric ligation, P1G10 inhibited pepsin activity by 69% in gastric juice^7^. Plant proteases due to their non-specific nature may cleave substrates (without activation) at sites different compared to the cognate inflammatory enzyme. It has been demonstrated for bromelain that selective removal of cell surface attractants (CD128) prevents neutrophil infiltration (*i*.*e*. inflammation)^36^. In this context, an indirect proteolytic inhibition strategy proposed as therapy for IBD^34^ fits within the role for plant proteolytic enzymes proposed here.

To evaluate the proteolytic role on mucosal damage, the fraction (0.3 mg/Kg) was proteolytically inactivated by the alkylating agent IAA (P1G10-IAA). The assay of the inhibited fraction shows that proteolytic activity is necessary for effectiveness, (Figs. 2b–c and 3a,b). In excisional lesions, P1G10 reduced its healing capacity when the proteolytic activity was inhibited^8^. Similarly, oral administration of active bromelain was required for efficacy as anti-inflammatory^37,38^. There is evidence that at least part of the anti-inflammatory activity of P1G10 hinges on its proteolytic activity. In murine 4T1 inflammatory breast carcinoma, modulation of the inflammatory process is influenced by the proteolytic activity of P1G10 (unpublished observation). Conversely, the proteolytic activity of P1G10 was not essential for gastric protection in indomethacin induced lesions^7^. These results suggest that some actions involving P1G10 entail the proteolytic activity while others do not. This apparent contradiction was answered following purification of the isoforms. When probing the mitogenic activity of isoforms CMS2MS2 and CMS2MS3, we found that activation of MAPK mitogenic pathway in L929 fibroblast did not required the proteolytic activity^39,40^. In sum, 2 out of 14 proteinases isoforms exhibited a mitogenic property independent of the proteolytic activity. In a similar example, a cysteine protease in plerocercoid *Spirometra mansonoides* displays growth hormone-like property unrelated to its proteolytic activity^41^.

The anti-inflammatory activity of P1G10 is associated to a reduction of MPO activity, suggesting a lower presence and/or activation of neutrophils (Fig. 4a). The decrease in neutrophil activation by P1G10 might be explained as demonstrated for bromelain by selective removal of neutrophil cell receptor, thus preventing inflammation^36^. Furthermore P1G10 prevents depletion of reduced glutathione (GSH)^7^, an antioxidant free thiol playing a protective role in animals with gastric ulcer and TNBS induced colitis^42^. On the other hand, macrophage activation, measured by NAG activity, did not show difference between groups (Fig. 4b), probably due to the limited interval, insufficient to visualize infiltrating macrophages occurring during active IBD^43^.

Macrophages play a key role modulating the inflammatory response; they become activated via a classical activation response (CAM) involving macrophage M1, (Th-1 inflammatory response)^44^. The Th-1 inflammatory response in IBDs model is characterized by an imbalance between pro- and anti-inflammatory cytokines, evidenced by increase in IL-1, IL-6, TNF-α and IFN-γ secreted by leukocytes and reduction in IL-10 and IL-4^11,12^. Although several reports^45,46^ show an increase in TNF-α accompanying the inflammatory process, the moderate rise observed here was not statistically different from the Sham group. Nonetheless, the apparent increase of TNF-α in the TNBS group was suppressed by 0.3 mg/Kg P1G10, as shown in Fig. 5b.

Furthermore, 0.3 mg/Kg P1G10 increased IL-10 (Fig. 5c), an anti-inflammatory cytokine which inhibits antigen presentation and production of pro-inflammatory cytokines^11,13^. Interestingly, proteolytically active bromelain reduced spontaneous inflammatory symptoms in IL-10^−/−^ knockout mice^37^. P1G10 also reduced MCP-1 (CCL2) level (Fig. 5a), a known chemoattractant leukocyte that prevents severe inflammation^47^.

The decrease in cyclooxygenase 2 (COX-2) in the TNBS group treated with 0.3 mg/Kg P1G10 (Fig. 6) argues for an anti-inflammatory effect as well, as is known that COX-2 contributes to production of prostaglandin E2 and thromboxane B2 and that inhibitors of COX-2 decrease the inflammatory symptoms in IBD^16^. The decline in COX-2 was accompanied by an increase in secretory cells (goblet cells) (Fig. 8), that restored mucus production, at sites where the hapten induced lesions^15^.

A contribution to the anti-inflammatory effect is caused by reduction of metalloproteinases-2 (MMP-2) and-9 (MMP-9) (the latter not significant) in colon tissue of animals treated with 0.3 mg/Kg P1G10 (Fig. 7). MMPs are held as biomarkers in IBDs as they regulate epithelial barrier function, immune response, angiogenesis, fibrosis, and wound healing^48,49^. Both, expression and levels of MMP-9 are augmented in IBD patients and MMP-2 is up-regulated in a rat TNBS-induced colitis model like shown here^49^. The reduction of MMP-2 and MMP-9 (the latter not significant) in colon samples of treated animals (Fig. 7) confirm the protective effect induced by P1G10. In a similar rat model, expression of MMP-2 and TNF-α were increased by TNBS and both changes were reversed by administration of Mesalazine, a 5-aminosalicylic acid compound produced in the colon and used for treatment of patients with mild-to-moderate ulcerative colitis^17^. In our model, P1G10 exerts effect similar to Mesalazine although the decline in TNF-α was not significant.

Collectively, these data suggest that P1G10 may contribute to an efficacious reestablishment of pro and anti-inflammatory cytokine equilibrium following colitis induction. Future studies using this model must include IFN-γ, IL-1, IL-6, IL-17 and JNK, to evaluate the effect of P1G10 on these mediators.

In spite that IBD is considered an incurable symptom, in the past twenty years, therapeutic options made important advances following clinical introduction of anti TNF-α, methotrexate, azathioprine, antibiotics and other novel substances^50–52^. These agents along with a number of herbs are now recognized as alternatives for treatment of IBD^53^. One of these agents, bromelain (*A*. *comosis*) draws our attention because is a mixture of cysteine proteinases^37^, like P1G10. Given the biochemical similarities between bromelain and P1G10 and their proposed roles as antitumor, anti-inflammatory, immunomodulator, and healer, we suggest that these plant enzymes share pharmacological properties which can be used as alternative to treat symptoms associated with IBDs.

## Conclusion

Proteolytically active P1G10 promotes resolution of lesions and restores the balance between pro-and anti-inflammatory modulators in TNBS induced colon lesions, attenuating the extent of injury in colonic tissue and colitis symptoms in mice. The data suggest that changes in IL-10 and MMP-2 are associated to the protective effect induced by P1G10.

## Supplementary information

Supplementary information.
