# Supplementary material for: The Proteolytic Fraction From Vasconcellea cundinamarcensis Latex Displays Anti-Inflammatory Effect in A Mouse Model of Acute TNBS-Induced Colitis
Source: Sci Rep. 2020 Feb 20;10:3074. doi: 10.1038/s41598-020-59895-3 (PMC7033115; doi:10.1038/s41598-020-59895-3)
Supplement: Supplementary file 1 — Supplementary information. [file 41598_2020_59895_MOESM1_ESM.pdf]

Supplementary information for “The proteolytic fraction from *Vasconcellea cundinamarcensis* latex displays anti-inflammatory effect in a mouse model of acute TNBS-induced colitis”

Ronniel Morais Albuquerque, Marina Passos Pizzitola, Ana Cândida Araújo e Silva, Dalton Dittz, Kátia Michelle Freitas, Ênio Ferreira, Carlos Edmundo Salas, Miriam Teresa Paz Lopes

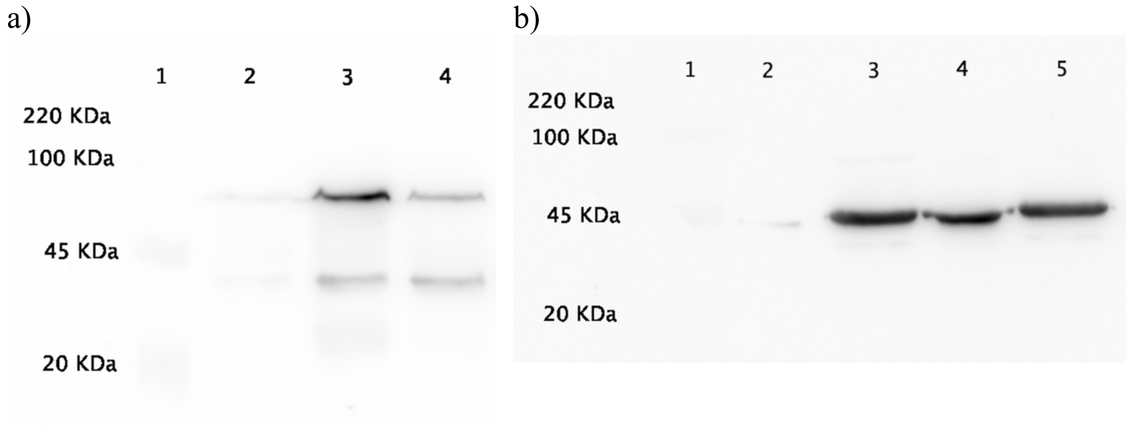

Supplementary Figure S1: Individual blots for COX-2 and β-actin. (a) Blot for COX-2 showing molecular weight standard, Sham, TNBS and P1G10 0.3 mg/Kg in columns 1-4, respectively; (b) Blot for β-actin showing molecular weight standard, none, sham, TNBS and P1G10 0.3 mg/Kg in columns 1-5, respectively. Excerpts from (a) and (b) were used in Fig. 6.

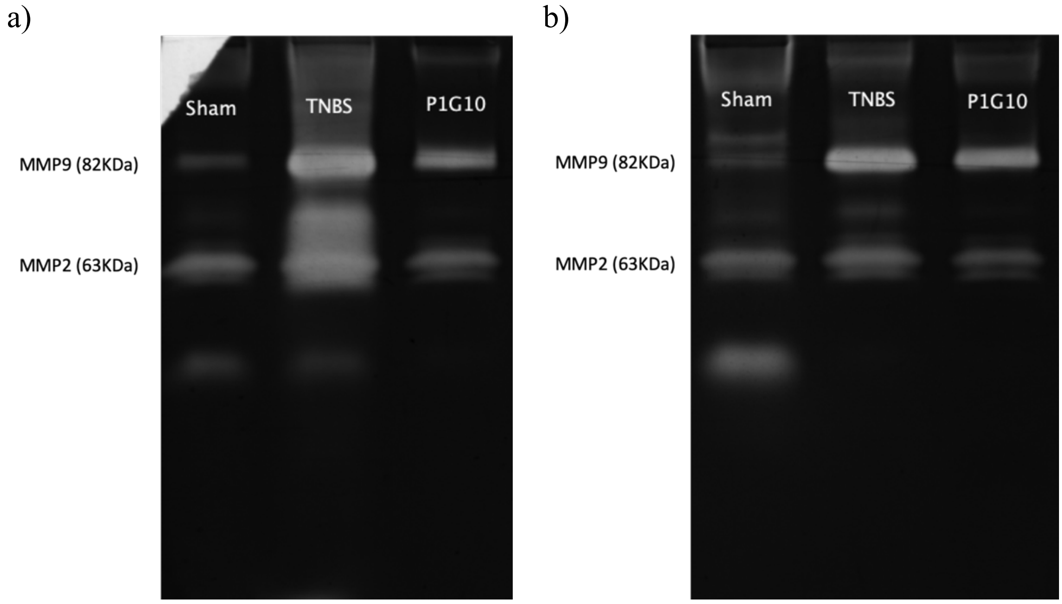

Supplementary Figure S2: Individual gels for MMP-9 and MMP-2. (a) Gel containing MMP-2 excerpt used in Fig. 7; (b) Gel containing MMP-9 excerpt used in Fig. 7.
